# *Hukou* status and perinatal depression: a longitudinal cohort study in China

**DOI:** 10.3389/fpubh.2025.1711901

**Published:** 2025-12-03

**Authors:** Tingting Zhou, Ying Sun, Fangyuan Chen, Xiuping Yang, Chen Jiang, Xuejun Gu

**Affiliations:** 1 Department of Women's Health Care, Women and Children's Hospital of Ningbo University, Ningbo, Zhejiang, China; 2 Department of Child Health Care, Women and Children's Hospital of Ningbo University, Ningbo, Zhejiang, China

**Keywords:** *hukou* status, perinatal depression, Edinburgh postnatal depression scale, socioeconomic status, pregnancy stages

## Abstract

**Objectives:**

Perinatal depression (PND) is a significant public health concern, particularly among those without local household registration. This study aims to investigate the association between China’s *hukou* status (household registration system) and PND, and to assess whether socioeconomic status (SES) modifies this relationship.

**Methods:**

A prospective cohort study was conducted among 1,825 pregnant women at four maternal and child health hospitals in Ningbo, China, from January to December 2022. Depressive symptoms were assessed by the Edinburgh Postnatal Depression Scale (EPDS) during each trimester and postpartum. Socioeconomic status (SES) was assessed via a 0–5 composite score comprising educational level, household monthly income, and employment status. Generalized Estimating Equations (GEE) were used to evaluate the association between *hukou* status and PND (EPDS ≥ 9), and to examine potential effect modification by SES. All models were verified in parallel using logistic regression analyses. The robustness of the associations was examined in sensitivity analysis using alternative EPDS thresholds (≥10 and ≥13).

**Results:**

In our study, local *hukou* status was significantly associated with a lower prevalence of PND compared with non-local *hukou* status (adjusted odds ratio [aOR] = 0.30, 95% CI: 0.22–0.42; *p* < 0.001), with the strongest protective effect observed in the second trimester (aOR = 0.28, 95% CI: 0.20–0.38). However, the interaction between *hukou* status and time was not statistically significant (*p* > 0.05). Interaction analyses between *hukou* status and SES further confirmed that the protective effect of local *hukou* was independent of SES (*p* > 0.05). Among the 1,825 pregnant women included, 345 (18.9%) held non-local *hukou*, and the prevalence of depression was 285 (15.62%), 237 (12.99%), 257 (14.08%), and 167 (9.15%) cases in the first, second, third trimesters, and postpartum period, respectively. Sensitivity analyses verified the robustness of all aforementioned findings.

**Conclusion:**

China’s *hukou* status functions as an independent institutional determinant of PND risk, with its protective effect unmodified by individual socioeconomic resources. Addressing the *hukou*-based institutional inequities is essential for developing effective public health strategies and clinical interventions against PND.

## 1 Introduction

Perinatal depression (PND), encompassing both major and minor depressive episodes occurring during pregnancy or within the first year postpartum, is one of the most prevalent medical complications in the perinatal period. The American College of Obstetricians and Gynecologists (ACOG) indicates that this condition affects approximately one in seven women during the period (1). While in China, relevant evidence highlights a comparable or slightly higher burden. For instance, a meta-analysis focusing on Chinese populations reported an overall PND prevalence of 16.3% (95% CI: 14.7–18.2%), with prenatal and postpartum rates reaching 19.7 and 14.8%, respectively (2). Globally, rural areas report a higher prevalence (22.1, 95% CI: 19.0–25.3%), and rates are further elevated in low- and lower-middle-income countries, at 24.5 and 22.8%, respectively (3). PND is a major public health issue with serious consequences for mothers and children. Depressed mothers face increased risks of obstetric complications, including preeclampsia (4) and gestational diabetes (5), with particularly heightened suicide risk (6, 7). Their infants are more likely to experience preterm birth (8), low birth weight, impaired growth, and psychomotor developmental delays (9). Beyond health impacts, PND also disrupts family relationships and creates economic burdens for households and society (10).

Beyond individual-level factors, perinatal mental health is profoundly shaped by structural social determinants. In China, the household registration (*hukou*) system institutionalizes such structural inequality by tying access to resources and opportunities to one’s registration locale (11, 12). This system classifies individuals as rural or urban residents, serving as a key socioeconomic indicator (13). The system aims to use administrative registration to anchor the population in specific geographic areas and restrict internal population movement (14). The *hukou* system can influence health outcomes through various pathways, including the allocation of medical resources and social security. In recent years, with economic development, the number of non-local workers in economically developed regions has increased. However, the actual social security and medical services they receive may fall below the standards enjoyed by local residents (13, 15, 16). Guo et al. found that both temporary and permanent non-local residents had significantly higher levels of depressive symptoms compared with urban local residents (14). Similarly, a nationwide survey by Qiu et al. revealed that non-local workers had a higher prevalence of depressive symptoms (23.7%), and the risk of depression was affected by socioeconomic status (SES) (17).

Existing studies have primarily identified several risk factors for PND, including premenstrual syndrome, violence exposure, unintended pregnancy (18), poor social support (19), low economic status of families, and low education level (20). However, few studies have explored the role of *hukou* status in PND in China, particularly its longitudinal effects across pregnancy stages. Moreover, higher SES (e.g., education, income) may buffer the adverse effects of disadvantaged *hukou* status by providing greater resources and coping strategies, whereas lower SES may compound these disadvantages. This potential interaction effect of SES in this relationship warrants further investigation.

In this study, we conducted a prospective cohort study in Ningbo, China, aimed to: (1) quantify the association between *hukou* status and perinatal depressive symptoms across pregnancy stages, adjusting for key confounders; and (2) assess whether SES modifies these associations. Our findings will provide new insights into the role of *hukou* status in PND and inform both clinical screening protocols and social policies mitigating health inequities among non-local mothers.

## 2 Methods

### 2.1 Study design and participants

This study recruited pregnant women who received first-trimester prenatal care (<14 weeks of gestation) at four maternal and child health hospitals in Ningbo, China, from January 1st to December 31st, 2022. A total of 2,000 eligible women were enrolled, with 500 participants per site, after providing written informed consent. Follow-up continued until two months postpartum.

Participants were included if they met all of the following criteria: (1) gestational age <14 weeks at enrollment; (2) singleton pregnancy; (3) willingness to complete baseline surveys and psychological assessments; (4) no prior diagnosis of psychiatric disorders; and (5) ability to comply with study protocols. Exclusion criteria included: (1) pre-existing neurological or brain disorders; (2) physical inability to complete self-administered questionnaires or psychological scales; or (3) high mobility or uncertain delivery location.

The study protocol was approved by the Medical Ethics Committee of Ningbo Women and Children’s Hospital (NBFE-2025-KY-134, renewed from EC2022-029). This study was conducted in accordance with the principles of the Declaration of Helsinki. All participants signed the informed consent form.

### 2.2 Data collection

All participants underwent initial depression screening using the Edinburgh postnatal depression scale (EPDS) and completed baseline questionnaires during the first trimester (<14 weeks). Follow-up EPDS assessments were conducted at three additional time points: the second trimester (14–27^+6^ weeks), the third trimester (≥28 weeks), and the postpartum period (42 days to 2 months after delivery). Demographic and socioeconomic characteristics, specifically age, education, income, employment, and marital status, were assessed solely at the first-trimester baseline. As these factors are generally stable over the relatively short perinatal period, they were not re-evaluated in follow-up visits. All Questionnaires were collected by trained interviewers. Among the 2,000 initially enrolled participants, 1,825 (91.3%) completed all follow-up assessments and were included in the final analysis.

### 2.3 Covariate

Covariates included maternal age, educational level (high school or below, college or above), only child status, marital status (married, divorced/unmarried), current employment status (unemployed, freelance, full-time employed), household monthly income (<10,000 RMB, 10,000–20,000 RMB, ≥20,000 RMB), gravidity (1, 2–4, ≥5 pregnancies), infectious disease history (including hepatitis B, syphilis, and HIV), pre-pregnancy BMI (<18.5 kg/m^2^ [underweight], 18.5–24 kg/m^2^ [normal weight], or ≥24 kg/m^2^ [overweight/obese]), and whether the current pregnancy was planned.

### 2.4 Definition of variables

*Hukou* status refers to China’s official household registration system, which legally ties individuals’ access to public services to their registered location (21). In our study, it was categorized as local and non-local. SES was determined through a composite scoring system incorporating three key variables: educational level, employment status, and household monthly income (22). Educational level was scored on a 0–1 scale (0 = high school or less, 1 = college degree or above), while household monthly income was scored 0–2 (0 = <10,000 RMB, 1 = 10,000–20,000 RMB, 2 = ≥20,000 RMB) and employment status was likewise scored 0–2 (0 = unemployed, 1 = freelance, 2 = full-time), yielding a total SES score ranging from 0 to 5. Based on this composite score, participants were categorized into two distinct groups: those with low SES (SES scores 0 to 2) and those with high SES (SES scores > 2). The detailed scoring criteria and point assignments for each variable are provided in Supplementary Table 1.

### 2.5 Measurement of depressive symptoms

The Edinburgh postnatal depression scale (EPDS) was employed to evaluate depressive symptoms in perinatal women and has been proven to be a useful tool for both prenatal and postpartum screening in the Chinese population (23). The scale consists of 10 items, with a total score ranging from 0 to 30; higher scores indicate more severe depressive symptoms. The Chinese version of EPDS has demonstrated high internal consistency (Cronbach’s alpha = 0.84) and reliability (split-half reliability coefficient = 0.74) (24). Additionally, it has a well-validated cutoff score of 9/10 for depression screening (25, 26). In our study, maternal EPDS scores ≥ 9 were considered the presence of depression symptoms (27–29).

### 2.6 Statistical analysis

In the current study, descriptive statistics were used to summarize the baseline characteristics of the study population. Continuous variables were compared using independent samples t-tests or one-way analysis of variance (ANOVA), while categorical variables were analyzed using Pearson’s χ^2^ tests or Fisher’s exact tests.

The association between *hukou* status and perinatal depressive symptoms (EPDS ≥ 9) was primarily assessed using Generalized Estimating Equations (GEE) to account for the longitudinal correlation of repeated measures across the four time points. All models adjusted for covariates, including maternal age, education, pre-pregnancy BMI, only child, *hukou* status, marital status, employment, household monthly income, gravidity, HIV, syphilis, hepatitis B, and other infectious diseases, and planned pregnancy. To evaluate potential effect modification by SES, an interaction term between *hukou* status and the composite SES score was included in the GEE model. To verify the robustness of our primary findings, we conducted complementary analyses using separate multivariable logistic regression models for each perinatal period (trimesters 1–3 and postpartum).

Furthermore, sensitivity analyses were performed by re-running both the GEE and logistic regression models using alternative EPDS cutoffs (≥ 10 and ≥ 13). For logistic regression models with low event rates (<5%), we implemented Firth’s logistic method to address potential small-sample bias. Before conducting the multivariable regression analyses, we performed Hosmer-Lemeshow goodness-of-fit tests and assessed multicollinearity using variance inflation factors (VIF) to ensure model fit and stability.

All statistical analyses were performed using R (version 4.4.3; R Foundation for Statistical Computing, Vienna, Austria) and SAS (version 9.4; SAS Institute, Cary, NC). A two-tailed *p*-value <0.05 was considered statistically significant.

## 3 Results

### 3.1 Characteristics of participants

As depicted in Table 1, a total of 1,825 pregnant women were included in this longitudinal study, with an average age of 29.53 ± 4.14 years. The prevalence of depressive symptoms was 15.62% in the first trimester, 12.99% in the second trimester, 14.08% in the third trimester, and 9.15% postpartum.

**Table 1:** Baseline characteristics and univariate analysis of depression.

Characteristic	The first trimester			The second trimester			The third trimester			Postpartum		
	Depression	Non-depression	*P*	Depression	Non-depression	*P*	Depression	Non-depression	*P*	Depression	Non-depression	*P*
Total	285 (15.62)	1540 (84.38)		237 (12.99)	1588 (87.01)		257 (14.08)	1568 (85.92)		167 (9.15)	1658 (90.85)	
Age (years) mean (SD)	29.20 (4.43)	29.60 (4.08)	0.137	29.15 (4.42)	29.59 (4.09)	0.123	29.50 (4.64)	29.54 (4.05)	0.889	28.55 (4.01)	29.63 (4.14)	**0.001**
Education			**<0.001**			**<0.001**			**<0.001**			**<0.001**
High school or below	122 (24.16)	383 (75.84)		103 (20.40)	402 (79.60)		96 (19.01)	409 (80.99)		83 (16.44)	422 (83.56)	
College or above	163 (12.35)	1157 (87.65)		134 (10.15)	1186 (89.85)		161 (12.20)	1159 (87.80)		84 (6.36)	1236 (93.64)	
*Hukou* status			**<0.001**			**<0.001**			**<0.001**			**<0.001**
Local	182 (12.30)	1298 (87.70)		133 (8.99)	1347 (91.01)		172 (11.62)	1308 (88.38)		95 (6.42)	1385 (93.58)	
Non-local	103 (29.86)	242 (70.14)		104 (30.14)	241 (69.86)		85 (24.64)	260 (75.36)		72 (20.87)	273 (79.13)	
Marital status			0.296*			0.771*			0.258*			1.000*
Married	279 (15.52)	1519 (84.48)		233 (12.96)	1565 (87.04)		251 (13.96)	1547 (86.04)		165 (9.18)	1633 (90.82)	
Divorced/unmarried	6 (22.22)	21 (77.78)		4 (14.81)	23 (85.19)		6 (22.22)	21 (77.78)		2 (7.41)	25 (92.59)	
Employment status			0.559			0.204			0.051			0.170
Full-time	183 (15.06)	1032 (84.94)		151 (12.43)	1064 (87.57)		159 (13.09)	1056 (86.91)		105 (8.64)	1110 (91.36)	
Freelance	51 (15.94)	269 (84.06)		39 (12.19)	281 (87.81)		44 (13.75)	276 (86.25)		27 (8.44)	293 (91.56)	
Unemployed	51 (17.59)	239 (82.41)		47 (16.21)	243 (83.79)		54 (18.62)	236 (81.38)		35 (12.07)	255 (87.93)	
Household monthly income			0.483			0.780			0.288			0.425
<10000RMB	106 (16.69)	529 (83.31)		85 (13.39)	550 (86.61)		100 (15.75)	535 (84.25)		56 (8.82)	579 (91.18)	
10,000-20000RMB	134 (15.55)	728 (84.45)		107 (12.41)	755 (87.59)		111 (12.88)	751 (87.12)		86 (9.98)	776 (90.02)	
≥20000RMB	45 (13.72)	283 (86.28)		45 (13.72)	283 (86.28)		46 (14.02)	282 (85.98)		25 (7.62)	303 (92.38)	
Gravidity			**0.008**			0.252			**0.013**			0.078
1	96 (12.66)	662 (87.34)		87 (11.48)	671 (88.52)		89 (11.74)	669 (88.26)		63 (8.31)	695 (91.69)	
2–4	168 (17.34)	801 (82.66)		135 (13.93)	834 (86.07)		147 (15.17)	822 (84.83)		89 (9.18)	880 (90.82)	
≥5	21 (21.43)	77 (78.57)		15 (15.31)	83 (84.69)		21 (21.43)	77 (78.57)		15 (15.31)	83 (84.69)	
SES			**0.004**			**0.007**			**0.018**			**0.038**
High	184 (14.09)	1122 (85.91)		152 (11.62)	1154 (88.36)		168 (12.86)	1138 (87.14)		108 (8.27)	1198 (91.73)	
Low	101 (19.46)	418 (80.54)		85 (16.38)	434 (83.62)		89 (17.15)	430 (82.85)		59 (11.37)	460 (88.63)	

SES: socioeconomic status. Data are presented as mean ± standard deviation (SD) (continuous variables) and frequencies (percentages) (categorical variables). T-test for continuous variables. Pearson’s χ^2^ or Fisher’s exact tests for categorical variables. *P* < 0.05 was set as the threshold of statistical significance and marked in bold values. BMI, body mass index. ^*^Fisher’s exact tests.

Significant differences (*p* < 0.05) in basic demographic data were observed between depressed and non-depressed women in the first trimester, including educational level, only-child status, *hukou* status, gravidity, infectious diseases (HIV, syphilis, hepatitis B, and others), pregnancy planning status, and SES. In the second trimester, significant differences (*p* < 0.05) were found in educational level, only-child status, *hukou* status, infectious diseases, and SES. In the third trimester, significant associations (*p* < 0.05) were identified in educational level, only-child status, *hukou* status, gravidity, infectious diseases, and SES. Postpartum assessments revealed significant differences (*p* < 0.05) in maternal age, educational level, only-child status, *hukou* status, infectious diseases, pregnancy planning status, and SES. The complete results are presented in Supplementary Table 2.

### 3.2 Depressive symptoms

**Figure 1:**
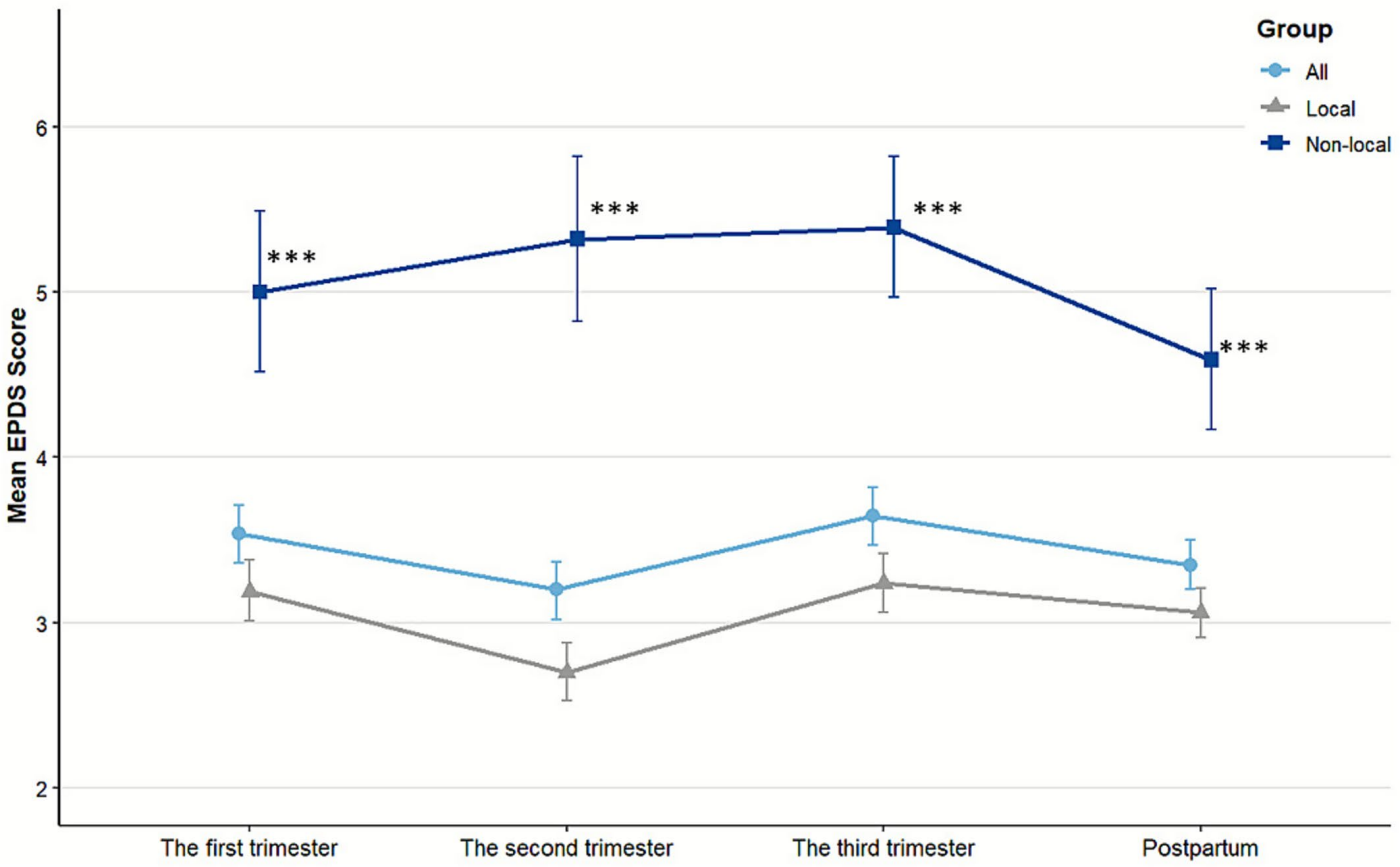
Mean EPDS scores with 95% confidence intervals by pregnancy stages and *hukou* status. Non-local women demonstrated significantly higher depression scores than local women at all stages (all *p* < 0.001). Significance levels: ^***^p < 0.001.

**Figure 2:**
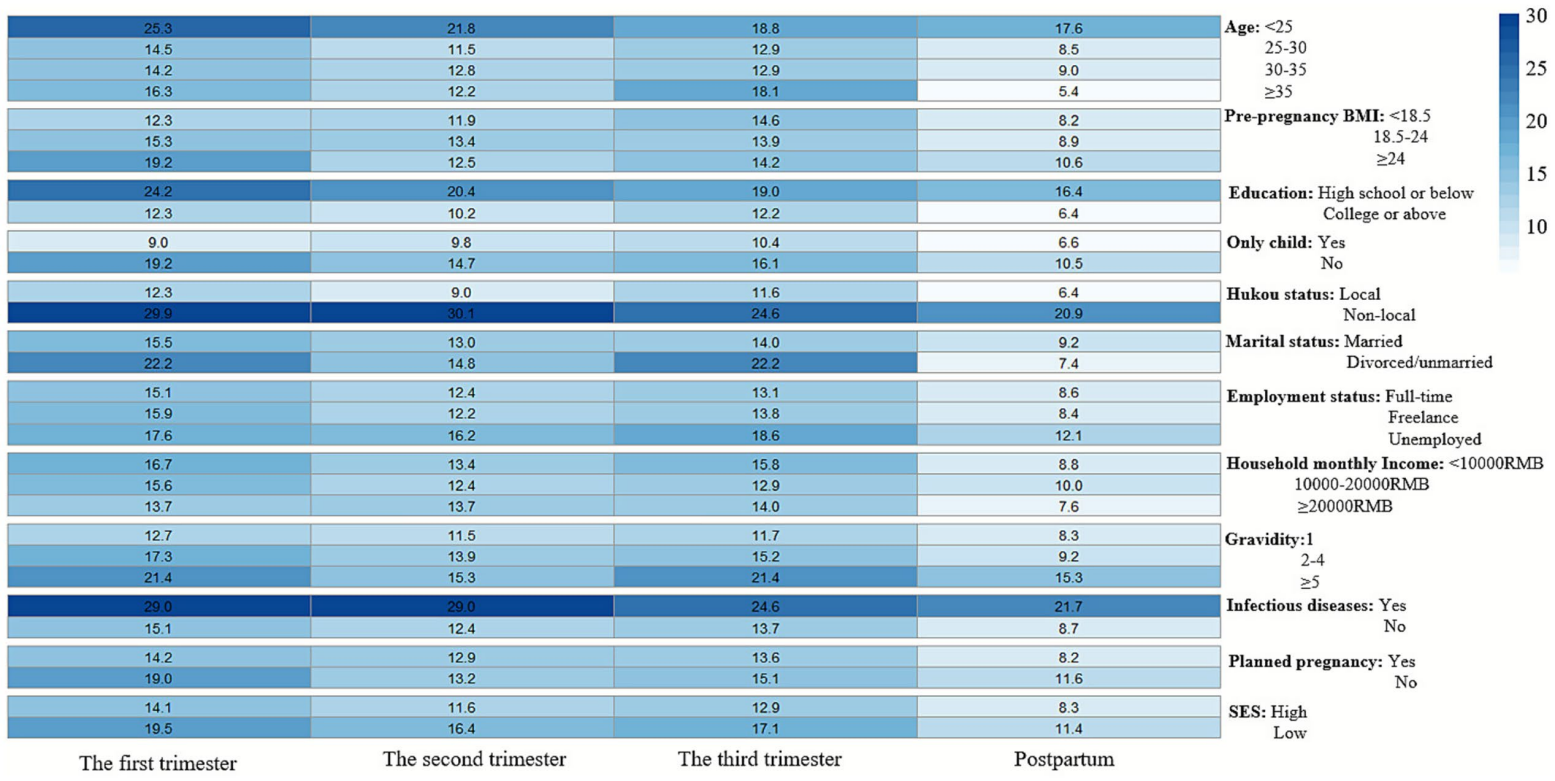
Heatmap of depression symptom prevalence (EPDS ≥ 9). Darker shading indicates higher incidence rates. The overall depression prevalence differences across pregnancy stages were statistically significant (*p* < 0.001).

Figures 1, 2 illustrate the longitudinal trajectories of depressive symptoms across perinatal stages. In our cohort, 29.10% (531/1,825) of pregnant women exhibited at least one EPDS score ≥ 9 during the four assessed stages. The overall differences in EPDS scores across stages reached statistical significance (*p* < 0.001), with peak severity observed during the third trimester (mean EPDS score = 3.65, 95% CI: 3.47–3.82). A notable divergence emerged in the second trimester: while local women’s scores decreased (mean = 2.70, 95% CI: 2.53–2.88), scores for non-local women increased (mean = 5.32, 95% CI: 4.82–5.82). Non-local women consistently demonstrated higher EPDS scores than their local counterparts at every stage (all *p* < 0.001; Figure 1, detailed data in Supplementary Table 3). Statistical analysis of overall depression symptom prevalence rates similarly revealed significant variations across gestational periods (*p* < 0.001; Figure 2). As visually presented in Figure 2, which illustrates depression rates by maternal characteristics at each study timepoint, the prevalence of depressive symptoms showed a consistent decline during the postpartum period. Notably, non-local women exhibited persistently elevated rates (20.9–30.1% across stages), with the most pronounced disparity occurring in the second trimester (30.1% vs. 9.0% for local women). Overall, the risk of depression for non-local women was consistently higher, with prevalence ratios of 2.43 in the first, 3.34 in the second, 2.12 in the third trimester, and 3.27 postpartum compared to their local counterparts.

### 3.3 Associations of depressive symptoms with *hukou* status and covariates across pregnancy stages

**Figure 3:**
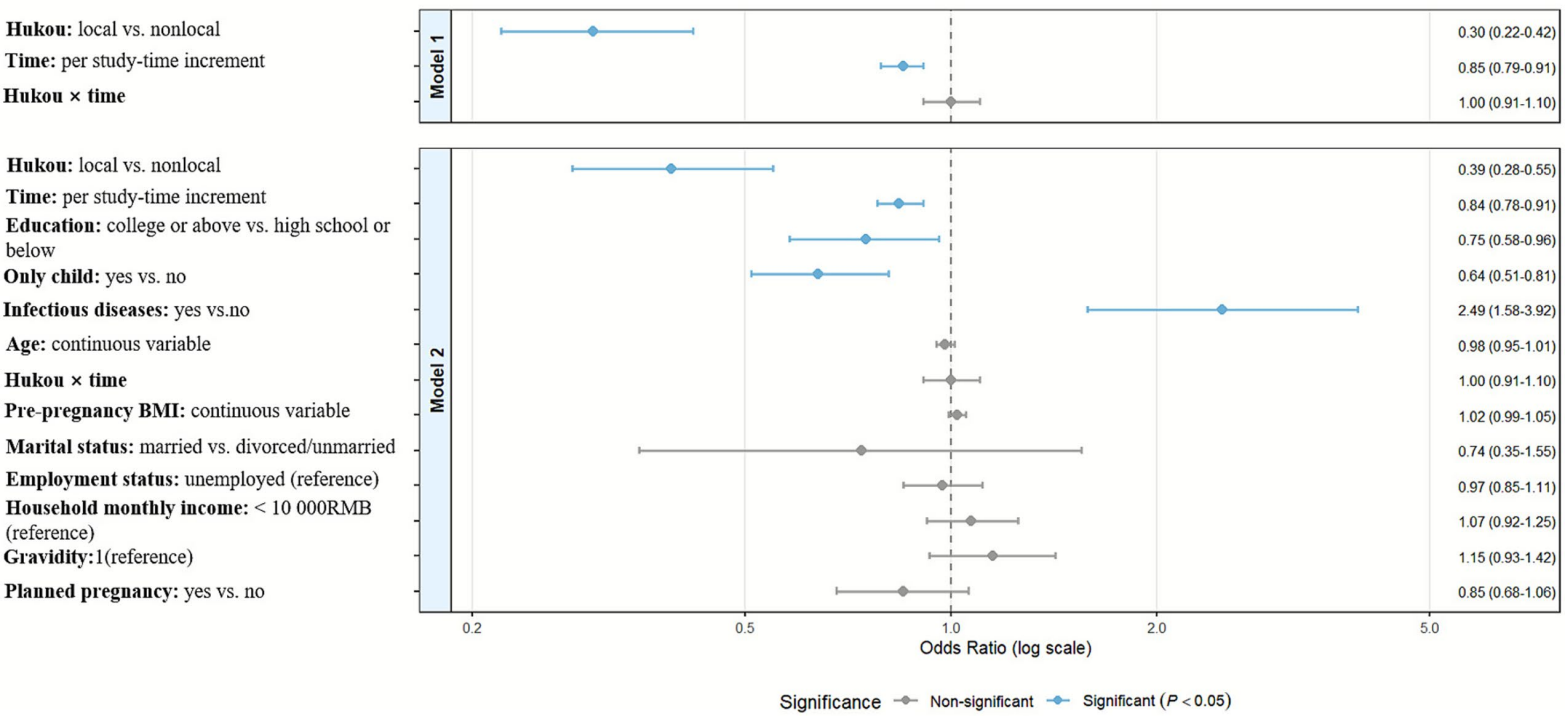
Forest plot analysis of *hukou* status and covariates associated with perinatal depression symptoms.

As shown in Table 2, the GEE analysis demonstrated that local *hukou* status conferred a significant protective effect against PND (aOR = 0.30, 95% CI: 0.22–0.42, *p* < 0.001), which remained robust after full adjustment for covariates (aOR = 0.39, 95% CI: 0.28–0.55, *p* < 0.001). With each successive study time point (from the first trimester to postpartum), the odds of depressive symptoms significantly decreased by 16% (aOR = 0.84, 95% CI: 0.78–0.91, *p* < 0.001) in the fully adjusted model (Model 2). Critically, the interaction between *hukou* status and time was not statistically significant (aOR = 1.00, 95% CI: 0.91–1.10, *p* = 0.995). This indicates that the protective effect of local *hukou* against depressive symptoms remained consistent in magnitude across all perinatal stages (trimesters 1–3 and postpartum) and did not vary significantly over time. Additionally, high degree (university and above), only-child status were found to be associated with a lower likelihood of depression (all aOR < 1, all *p* < 0.05), while history of infectious diseases was associated with increased PND risk (aOR = 2.49, 95% CI: 1.58–3.92, *p* < 0.001). These findings are visually summarized in Figure 3. Supplementary multivariable logistic regression analyses, which demonstrated adequate model fit (Hosmer-Lemeshow test *p* > 0.05, Supplementary Table 4) and no concerning multicollinearity (VIF < 2, Supplementary Table 5), consistently showed significantly reduced odds of depressive symptoms among women with local *hukou* across all perinatal stages compared to non-local women (all *p* < 0.05). Detailed results are provided in Supplementary Table 6 and Supplementary Figure 1.

**Table 2:** Association between *hukou* status (local vs. non-local) and perinatal depressive symptoms using GEE (*N* = 7,300 observations).

Characteristic	β	SE	Wald χ^2^	*P*	aOR	95%CI
Model 1						
*Hukou*: local vs. non-local	−1.190	0.167	50.9	**<0.001**	0.30	(0.22, 0.42)
Time: per study-time increment^a^	−0.168	0.037	21.1	**<0.001**	0.85	(0.79, 0.91)
*Hukou* × time	−0.002	0.049	0.00	0.960	1.00	(0.91, 1.10)
Model 2						
*Hukou*: local vs. non-local	−0.940	0.178	27.92	**<0.001**	0.39	(0.28, 0.55)
Time: per study-time increment	−0.173	0.038	21.19	**<0.001**	0.84	(0.78, 0.91)
Education: college or above vs. high school or below	−0.291	0.128	5.16	**0.023**	0.75	(0.58, 0.96)
Only child: yes vs. no	−0.445	0.117	14.45	**<0.001**	0.64	(0.51, 0.81)
HIV, syphilis, hepatitis B, and other infectious diseases: yes vs. no	0.912	0.234	15.24	**<0.001**	2.49	(1.58, 3.92)
Age: continuous variable	−0.021	0.014	2.11	0.147	0.98	(0.95, 1.01)
Pre-pregnancy BMI: continuous variable	0.019	0.016	1.44	0.230	1.02	(0.99, 1.05)
Marital status: married vs. divorced/unmarried	−0.302	0.382	0.63	0.429	0.74	(0.35, 1.55)
Employment status^b^	−0.034	0.069	0.24	0.624	0.97	(0.85, 1.11)
Household monthly income: < 10,000 (reference)^c^	0.072	0.077	0.87	0.350	1.07	(0.92, 1.25)
Gravidity: 1 (reference)^d^	0.141	0.108	1.72	0.190	1.15	(0.93, 1.42)
Planned pregnancy: yes vs. no	−0.164	0.114	2.07	0.150	0.85	(0.68, 1.06)
*Hukou* × time	0.000	0.050	0.00	0.995	1.00	(0.91, 1.10)

aOR = adjusted odds ratio; CI = confidence interval; GEE = Generalized Estimating Equations. *P* < 0.05 was set as the threshold of statistical significance and marked in bold values. Model 1 included *hukou* status, time and *hukou* × time as the variable. Model 2 adjusted for age, education, pre-pregnancy BMI, only child, marital status, employment, household monthly income, gravidity, HIV, syphilis, hepatitis B, and other infectious diseases, and planned pregnancy. Both models used an exchangeable working correlation structure. α = 0.328 (SE = 0.045) for Model 1 and α = 0.296 (SE = 0.047) for Model 2, indicating moderate within-subject correlation across the four perinatal visits.

a Time variable assignment: 1 = the first trimester of pregnancy, 2 = the second trimester of pregnancy, 3 = the third trimester of pregnancy, 4 = postpartum period.

b Employment status: 1 = unemployed (reference), 2 = freelance, 3 = full-time employed.

c Monthly household income (RMB): 1 = < 10,000 (reference), 2 = 10,000–20,000, 3 = ≥ 20000.

d Gravidity: 1 = gravidity 1 (reference), 2 = gravidity 2–4, 3 = gravidity ≥ 5.

### 3.4 Interaction analysis between *hukou* status and SES on depression risk

**Figure 4:**
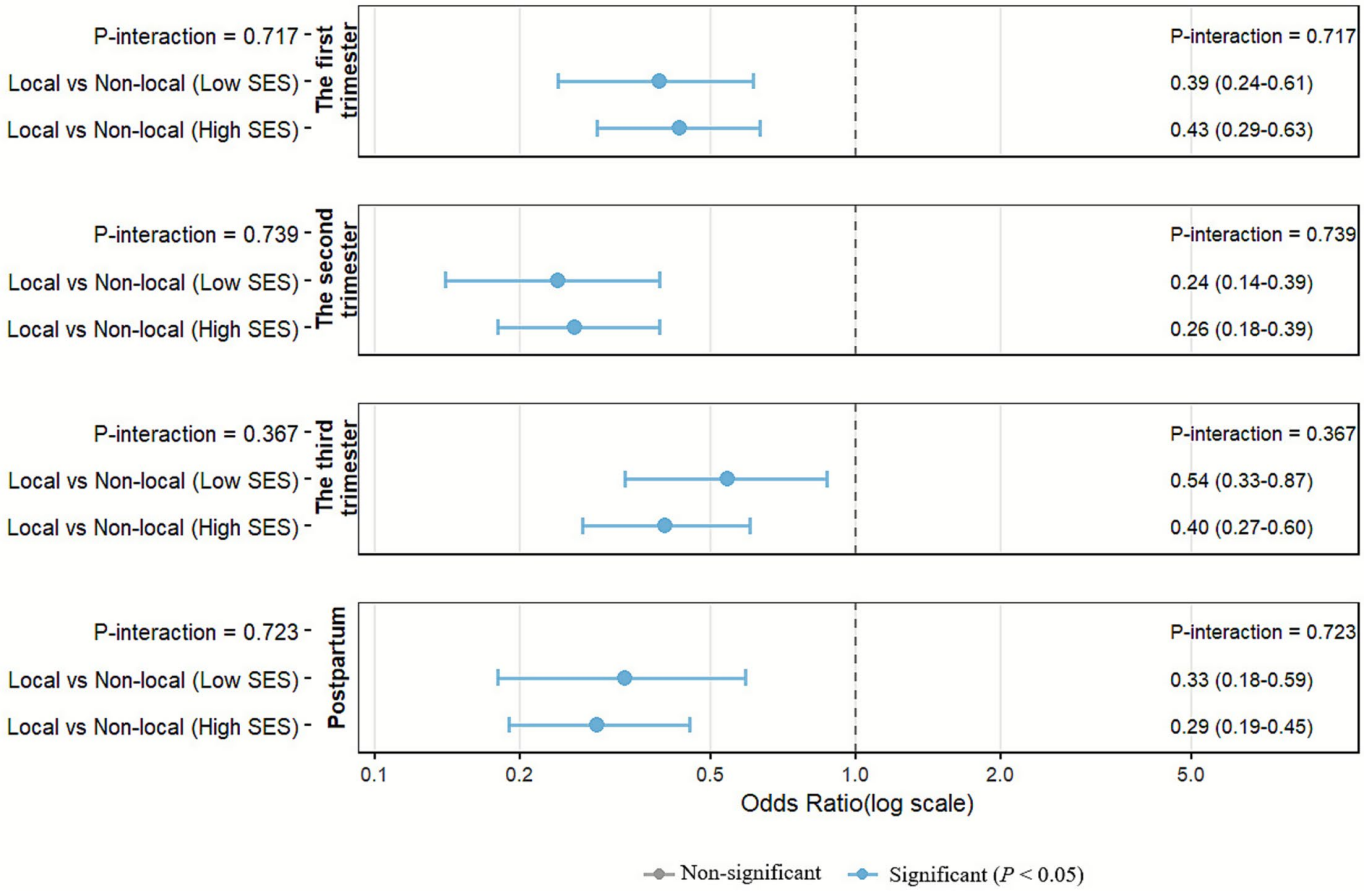
Interaction effects between *hukou* status and SES on depression risk across pregnancy stages (multivariate logistic regression). SES: socioeconomic status. All models adjusted for age, *hukou* status, SES, pre-pregnancy BMI, only child, marital status, gravidity, HIV, syphilis, hepatitis B, and other infectious diseases, and planned pregnancy.

Interaction results are presented in Table 3. In the longitudinal GEE model, the multiplicative interaction between *hukou* status and SES was non-significant both before and after adjustment for covariates (Model 1: aOR = 0.95, 95% CI 0.58–1.55, *P*-interaction = 0.832; Model 2: aOR = 0.97, 95% CI 0.60–1.57, *P*-interaction = 0.901). Furthermore, interactions between *hukou* status and each of the individual SES components (education, household monthly income, and employment status) also showed no statistically significant effects (all *P*-interaction > 0.05, Supplementary Table 7). Thus, we found no evidence that the protective effect of local *hukou* varies by SES level. To further explore these associations descriptively, we also conducted stratum-specific analyses using separate multivariable logistic regression models at each visit (Figure 4). The direction of effect was consistent (all *P*-interaction>0.05). Local *hukou* was associated with lower depression risk in both SES strata across all time points. Thus, the data support the conclusion that *hukou* status acts as a determinant of depression risk irrespective of SES.

**Table 3:** Effect of the *hukou* × SES interaction on perinatal depressive symptoms using GEE (*N* = 7,300 observations).

Characteristic	β	SE	Wald χ^2^	*P*	aOR	95% CI
Model 1						
*Hukou* × SES	−0.052	0.247	0.04	0.832	0.95	(0.58, 1.55)
Model 2						
*Hukou* × SES	−0.031	0.249	0.02	0.901	0.97	(0.60, 1.57)

aOR = adjusted odds ratio; CI = confidence interval, GEE = Generalized Estimating Equations. *P* < 0.05 was set as the threshold of statistical significance. Both models examine the *hukou* × SES interaction; Model 1 contains *hukou* status, time, SES, *hukou* × SES and *hukou* × time; Model 2 additionally adjusts for age, pre-pregnancy BMI, only-child status, marital status, gravidity, HIV, syphilis, hepatitis B, other infectious diseases, and planned pregnancy. Both models used an exchangeable working correlation structure. α = 0.324 (SE = 0.044) for Model 1 and Model 2, indicating moderate within-subject correlation across the four perinatal visits. Time variable assignment: 1 = the first trimester of pregnancy, 2 = the second trimester of pregnancy, 3 = the third trimester of pregnancy, 4 = postpartum period.

### 3.5 Sensitivity analysis

In our sensitivity analyses, the prevalence trends of depressive symptoms remained largely consistent across perinatal periods when using different EPDS cutoffs (≥ 9, ≥ 10, ≥ 13) (Supplementary Table 8). In the adjusted GEE model, local *hukou* remained a significant protective factor at both the EPDS ≥ 10 (aOR = 0.44, 95% CI: 0.30–0.63, *p* < 0.001) and EPDS ≥ 13 (aOR = 0.31, 95% CI: 0.14–0.65, *p* = 0.002) thresholds (Supplementary Table 9). These findings were further confirmed by supplementary logistic regression analyses, where the protective effect was uniformly observed in each perinatal period after full adjustment (for EPDS ≥ 10: aOR range 0.21–0.55, all *p* < 0.05; for EPDS ≥ 13: aOR range 0.12–0.39, all *p* < 0.05) (Supplementary Table 10).

## 4 Discussion

In this study, we found that the prevalence of depressive symptoms (EPDS ≥ 9) significantly decreased in the postpartum period. To the best of our knowledge, this is the first investigation of the longitudinal association between *hukou* status and PND across pregnancy and postpartum stages in Chinese women. The results suggested that China’s *hukou* system significantly influences PND risk, with local *hukou* conferring consistent protection across all pregnancy stages and postpartum. Notably, the nonsignificant interaction between *hukou* status and SES indicated that this protective effect was not modified by SES.

Our study found that the prevalence of depression in the first trimester was 15.62%, while the incidence of postpartum depression symptoms decreased significantly (9.15%). This trend remained consistent even after stratification by basic demographic characteristics. This overall prevalence of PND in Ningbo is lower than rates reported in many other parts of China. For instance, a nationwide meta-analysis reported an overall PND prevalence of 16.3%, with prenatal and postpartum depression rates of 19.7% and 14.8%, respectively (30). Furthermore, studies from specific Chinese regions have reported higher rates, such as a postpartum depression prevalence of 23.5% in Shanghai (2019) (31) and 18.0% in Guangdong and Guangxi provinces (2021–2023) (32). Despite the lower overall prevalence in Ningbo, our study further reveals a stark disparity: the prevalence of depressive symptoms among non-local women persistently exceeded 20% across all perinatal stages. This rate is comparable to the general prevalence reported among pregnant women in other Chinese regions but is significantly higher than that of their local counterparts within Ningbo. Globally, a systematic review showed that the average overall prevalence rates of PND, prenatal depression, and postpartum depression were 26.3%, 28.5%, and 27.6%, respectively (33). Another meta-analysis revealed that the overall prevalence of PND was 24.7% (95% CI: 23.7–25.6%). The highest prevalence was observed in low- and middle-income countries, with an overall prevalence of 25.5% (95% CI: 23.8–27.1%), followed by upper-middle-income countries (overall prevalence of 24.7, 95% CI: 23.6–25.9%). Additionally, the prevalence of PND was lowest in the East Asia and Pacific region, at 21.4% (95% CI: 19.8–23.1%) (34). Our EPDS-based postpartum depression rates in Ningbo were significantly lower than those in Italy (35) and Ethiopia (19).

In the current study, the incidence of depressive symptoms among non-local registered pregnant women in Ningbo was significantly higher than that among local registered pregnant women (*p* < 0.05). We found that even after adjusting for covariates such as age, pre-pregnancy BMI, and education level, local *hukou* status remained a significant protective factor against perinatal depressive symptoms (aOR = 0.39, 95% CI: 0.28–0.55, *p* < 0.001). This protective association was consistent over time and remained statistically significant throughout all stages of pregnancy and the postpartum period (*p* < 0.001). Since China’s *hukou* system directly affects access to healthcare, housing eligibility, and educational resources (16), it is closely related to perinatal health. A study conducted in Beijing revealed that the non-local population has lower insurance coverage and utilization rates (36). A large-scale study in Shanghai further demonstrated that the unique *hukou* system restricts internal migrant women’s access to public services, thereby creating a potential pathway to the observed increased risk of high-risk pregnancies in this population (37). It is closely related to perinatal health. Our findings align with those of other international studies on immigrant populations (38, 39). Additionally, research indicates that the psychological status of rural and urban migrants is poor and merits further investigation (40). Thus, China’s unique *hukou* system deprives internally non-local women of public health services and social support compared to local registered women, rendering them a potentially vulnerable group. Taken together, our core finding is that the *hukou* system is an independent determinant of perinatal mental health—and this likely points to a systemic risk that exists nationwide. While the manifestation and intensity of this risk may vary across regions, it is reasonable to argue that the effect could be even more pronounced in areas with fewer social safety nets.

SES, including education level and family economic status, is an important factor contributing to maternal depressive symptoms (20, 41). Our interaction analysis shows that the protective effect of *hukou* status exists independently of SES. Past studies suggest that high SES may reduce the risk of depression among immigrant populations. A meta-analysis further underscores the vulnerability of this group, revealing that one in four migrant women develops PND, with the burden appearing even higher among forced migrants compared to economic migrants (42). Supporting this, a study in Belgium reported that immigrants from Turkey and Morocco had significantly higher prevalence of depressive symptoms compared to native Belgians (20.9% vs. 5.85–9.43%). Additionally, the study’s multivariate analysis suggested that high SES may mitigate the risk of depressive symptoms (43). However, a survey in Spain found that the self-reported prevalence of depression and antidepressant use among immigrant groups was lower than among native Spanish adults (44). This may be attributed to the healthy immigrant effect (45), as immigrants who remained in the country after the economic crisis had more favorable conditions in terms of social status and health. Similarly, studies in China have shown that the level of depressive symptoms is significantly higher among the temporary non-local population (*β* = 0.97, 95% CI: 0.38–1.56) and the permanent non-local population (β = 0.52, 95% CI: 0.04–1.00) compared to urban residents (14). This study demonstrates that China’s *hukou* system may limit the conversion of socio-economic resources into mental health benefits for non-local women, thereby making it challenging for them to achieve the same level of psychological protection even if they possess a higher economic status (46). Psychological interventions for the non-local population need to go beyond mere economic assistance and focus on fundamental reforms to the restrictive *hukou* system and the uneven distribution of resources and opportunities in urban and rural areas (40).

Compared to international migration studies, China presents a distinct paradigm. International literature often attributes deteriorating immigrant mental health primarily to language barriers, cultural differences, and workplace discrimination (47, 48). In China, however, the *hukou* system serves as a powerful structural determinant, legally tying access to public services and welfare benefits to one’s registered residence (49). This direct institutional barrier may prove more fundamental and intractable than the sociocultural obstacles faced by international migrants. The vulnerability of non-local women manifests through multiple pathways: institutional barriers to healthcare access; weakened social support networks due to migration; and socioeconomic insecurity. The paradoxical lack of a protective effect from higher SES further suggests that *hukou*-related implicit barriers, such as unequal access to quality resources, may counteract the benefits typically conferred by socioeconomic advantages. To address this structural unfairness, we recommend two pathways to delink medical service access from the *hukou* system: Firstly, enhance the existing residence permit system by explicitly granting non-local resident equal access to basic public services, including prenatal care. Secondly, accelerate the development of a nationwide, interoperable electronic maternal health record to eliminate information gaps and duplicate testing across regions. Clinically, these findings call for integrating *hukou* status into routine psychosocial assessments. Given the persistent risk, we recommend repeated depression screening for non-local women throughout the entire perinatal period, coupled with proactive referrals to community support resources.

This study has several strengths. First, it employs a prospective cohort design, with consecutive recruitment from four regional maternal and child health institutions, thereby enhancing the study’s internal validity. Second, longitudinal assessment of depressive symptoms across all pregnancy trimesters and postpartum provides dynamic data superior to cross-sectional approaches. However, this study also has some limitations. First, because we did not measure potential mediators such as access to health care, social support, or perceived discrimination, we cannot identify the specific pathways through which local *hukou* exerts its protective effect. Second, our sample consisted mainly of married, employed women, which limits the generalizability of the findings to more vulnerable non-local groups (e.g., unmarried women or those without stable employment). Third, although the EPDS is well suited for large-scale epidemiologic screening and our sensitivity analyses confirmed the robustness of the results, the absence of a clinical interview to confirm diagnoses may have reduced the clinical specificity of our findings. Finally, as an observational study, residual confounding cannot be ruled out despite adjustment for a wide range of covariates and extensive sensitivity analyses. Future research should incorporate the aforementioned mediators, deliberately recruit more vulnerable sub-populations, and integrate clinical diagnoses to further validate and extend the present findings.

## 5 Conclusion

In conclusion, this study demonstrates that China’s *hukou* system remains significantly associated with PND after adjustment for measured confounders, with local *hukou* providing substantial protection across all pregnancy stages. Therefore, decoupling healthcare access from *hukou* status and implementing targeted interventions during high-risk periods may help alleviate mental health disparities among China’s non-local pregnant population.

## Data Availability

The datasets presented in this article are not readily available due to privacy and ethical restrictions concerning research involving pregnant women; the raw data are not deposited in a public repository. Requests to access the datasets should be directed to XG, at xuejungunb@163.com.
