# Supplementary material for: Hukou status and perinatal depression: a longitudinal cohort study in China
Source: Front Public Health. 2025 Dec 3;13:1711901. doi: 10.3389/fpubh.2025.1711901 (PMC12708236; doi:10.3389/fpubh.2025.1711901)
Supplement: Supplementary file 1 [file Data_Sheet_1.docx]

Supplementary Material

**Content**

**Supplementary Table 1** Socioeconomic status measure**2**

**Supplementary Table 2** Baseline characteristics and univariate analysis of depression.**3**

**Supplementary Table 3** Mean EPDS scores with 95% confidence intervals by pregnancy stage and *hukou* status **5**

**Supplementary Table 4** Hosmer-Lemeshow Goodness-of-Fit Test results for logistic regression models across different gestational and postpartum stages**5**

**Supplementary Table 5** Results of multicollinearity analysis (variance Inflation Factor, VIF) **5**

**Supplementary Table 6** Multivariate logistic regression of depressive symptoms (EPDS≥9) related to hukou status and sociodemographic factors across pregnancy stages**6**

**Supplementary Table 7** Longitudinal GEE analyses of three hukou-based SES interactions on perinatal depressive symptoms (N = 7,300 observations) **8**

**Supplementary Table 8** Incidence of depressive symptoms across perinatal stages under different EPDS cutoff values**8**

**Supplementary Table 9** Longitudinal GEE analyses of the association between hukou status and perinatal depression under different EPDS cutoff values (N = 7,300 observations) **8**

**Supplementary Table 10** Logistic regression analysis of the association between hukou status and perinatal depression under different EPDS cutoff values **9**

**Supplementary Fig. 1** Forest plot analysis of *hukou* status and covariates associated with depression symptoms across pregnancy stages (multivariate logistic regression) **10**

**Supplementary Table 1** Socioeconomic status measure*

| **Socioeconomic Status** | **Score** |
| --- | --- |
| **Family monthly income** |  |
| <10000 RMB | 0 |
| 10000-20000 RMB | 1 |
| ≥20000 RMB | 2 |
| **Education** |  |
| High school or less | 0 |
| College degree or above | 1 |
| **Employment Status** |  |
| unemployed | 0 |
| Freelance | 1 |
| Full-time | 2 |

Note: * The aggregate score was used to classify participants into low socioeconomic status (SES scores 0 to 2), or high socioeconomic status (SES scores>2).

**Supplementary Table 2** Baseline characteristics and univariate analysis of depression.

|  | The first trimester | |  | The second trimester | |  | The third trimester | |  | Postpartum | |  |
| --- | --- | --- | --- | --- | --- | --- | --- | --- | --- | --- | --- | --- |
|  | Depression | Non-  depression | *P* | Depression | Non-depression | *P* | Depression | Non-depression | *P* | Depression | Non-depression | *P* |
| Total | 285(15.62) | 1540(84.38) |  | 237(12.99) | 1588(87.01) |  | 257(14.08) | 1568(85.92) |  | 167(9.15) | 1658(90.85) |  |
| Age (years) Mean (SD) | 29.20(4.43) | 29.60(4.08) | 0.137 | 29.15(4.42) | 29.59(4.09) | 0.123 | 29.50(4.64) | 29.54(4.05) | 0.889 | 28.55(4.01) | 29.63(4.14) | **0.001** |
| Pre-pregnancy BMI |  |  | 0.053 |  |  | 0.791 |  |  | 0.963 |  |  | 0.537 |
| <18.5 kg/m^2^ | 33(12.31) | 235(87.69) |  | 32(11.94) | 236(88.06) |  | 39(14.55) | 229(85.45) |  | 22(8.21) | 246(91.79) |  |
| 18.5-24 kg/m^2^ | 183(15.28) | 1015(84.72) |  | 160(13.36) | 1038(86.64) |  | 167(13.94) | 1031(86.06) |  | 107(8.93) | 1091(91.07) |  |
| ≥24 kg/m^2^ | 69(19.22) | 290(80.78) |  | 45(12.53) | 314(87.47) |  | 51(14.21) | 308(85.79) |  | 38(10.58) | 321(89.42) |  |
| Education |  |  | **<0.001** |  |  | **<0.001** |  |  | **<0.001** |  |  | **<0.001** |
| High school or below | 122(24.16) | 383(75.84) |  | 103(20.40) | 402(79.60) |  | 96(19.01) | 409(80.99) |  | 83(16.44) | 422(83.56) |  |
| College or above | 163(12.35) | 1157(87.65) |  | 134(10.15) | 1186(89.85) |  | 161(12.20) | 1159(87.80) |  | 84(6.36) | 1236(93.64) |  |
| Only child |  |  | **<0.001** |  |  | **0.003** |  |  | **<0.001** |  |  | **0.006** |
| Yes | 57(8.98) | 578(91.02) |  | 62(9.76) | 573(90.24) |  | 66(10.39) | 569(89.61) |  | 42(6.61) | 593(93.39) |  |
| No | 228(19.16) | 962(80.84) |  | 175(14.71) | 1015(85.29) |  | 191(16.05) | 999(83.95) |  | 125(10.50) | 1065(89.50) |  |
| *Hukou* status |  |  | **<0.001** |  |  | **<0.001** |  |  | **<0.001** |  |  | **<0.001** |
| Local | 182(12.30) | 1298(87.70) |  | 133(8.99) | 1347(91.01) |  | 172(11.62) | 1308(88.38) |  | 95(6.42) | 1385(93.58) |  |
| Non-local | 103(29.86) | 242(70.14) |  | 104(30.14) | 241(69.86) |  | 85(24.64) | 260(75.36) |  | 72(20.87) | 273(79.13) |  |
| Marital status |  |  | 0.296* |  |  | 0.771* |  |  | 0.258* |  |  | 1.000* |
| Married | 279(15.52) | 1519(84.48) |  | 233(12.96) | 1565(87.04) |  | 251(13.96) | 1547(86.04) |  | 165(9.18) | 1633(90.82) |  |
| Divorced/unmarried | 6(22.22) | 21(77.78) |  | 4(14.81) | 23(85.19) |  | 6(22.22) | 21(77.78) |  | 2(7.41) | 25(92.59) |  |
| Employment status |  |  | 0.559 |  |  | 0.204 |  |  | 0.051 |  |  | 0.170 |
| Full-time | 183(15.06) | 1032(84.94) |  | 151(12.43) | 1064(87.57) |  | 159(13.09) | 1056(86.91) |  | 105(8.64) | 1110(91.36) |  |
| Freelance | 51(15.94) | 269(84.06) |  | 39(12.19) | 281(87.81) |  | 44(13.75) | 276(86.25) |  | 27(8.44) | 293(91.56) |  |
| Unemployed | 51(17.59) | 239(82.41) |  | 47(16.21) | 243(83.79) |  | 54(18.62) | 236(81.38) |  | 35(12.07) | 255(87.93) |  |
| Household monthly income |  |  | 0.483 |  |  | 0.780 |  |  | 0.288 |  |  | 0.425 |
| <10000RMB | 106(16.69) | 529(83.31) |  | 85(13.39) | 550(86.61) |  | 100(15.75) | 535(84.25) |  | 56(8.82) | 579(91.18) |  |
| 10000-20000RMB | 134(15.55) | 728(84.45) |  | 107(12.41) | 755(87.59) |  | 111(12.88) | 751(87.12) |  | 86(9.98) | 776(90.02) |  |
| ≥20000RMB | 45(13.72) | 283(86.28) |  | 45(13.72) | 283(86.28) |  | 46(14.02) | 282(85.98) |  | 25(7.62) | 303(92.38) |  |
| Gravidity |  |  | **0.008** |  |  | 0.252 |  |  | **0.013** |  |  | 0.078 |
| 1 | 96(12.66) | 662(87.34) |  | 87(11.48) | 671(88.52) |  | 89(11.74) | 669(88.26) |  | 63(8.31) | 695(91.69) |  |
| 2-4 | 168(17.34) | 801(82.66) |  | 135(13.93) | 834(86.07) |  | 147(15.17) | 822(84.83) |  | 89(9.18) | 880(90.82) |  |
| ≥5 | 21(21.43) | 77(78.57) |  | 15(15.31) | 83(84.69) |  | 21(21.43) | 77(78.57) |  | 15(15.31) | 83(84.69) |  |
| HIV, syphilis, hepatitis B, and other infectious diseases |  |  | **<0.002** |  |  | **<0.001** |  |  | **0.010** |  |  | **<0.001** |
| Yes | 20(28.99) | 49(71.01) |  | 20(28.99) | 49(71.01) |  | 17(24.64) | 52(75.36) |  | 15(21.74) | 54(78.26) |  |
| No | 265(15.09) | 1491(84.91) |  | 217(12.36) | 1539(87.64) |  | 240(13.67) | 1516(86.33) |  | 152(8.66) | 1604(91.34) |  |
| Planned pregnancy |  |  | **0.010** |  |  | 0.831 |  |  | 0.415 |  |  | **0.021** |
| Yes | 183(14.20) | 1106(85.80) |  | 166(12.88) | 1123(87.12) |  | 176(13.65) | 1113(86.35) |  | 105(8.15) | 1184(91.85) |  |
| No | 102(19.03) | 434(80.97) |  | 71(13.25) | 465(86.75) |  | 81(15.11) | 455(84.89) |  | 62(11.57) | 474(88.43) |  |
| SES |  |  | **0.004** |  |  | **0.007** |  |  | **0.018** |  |  | **0.038** |
| High | 184(14.09) | 1122(85.91) |  | 152(11.62) | 1154(88.36) |  | 168(12.86) | 1138(87.14) |  | 108(8.27) | 1198(91.73) |  |
| Low | 101(19.46) | 418(80.54) |  | 85(16.38) | 434(83.62) |  | 89(17.15) | 430(82.85) |  | 59(11.37) | 460(88.63) |  |

Note: SES: socioeconomic status. Data are presented as mean ± standard deviation (SD) (continuous variables) and frequencies (percentages) (categorical variables). T-test for continuous variables. Pearson's χ² or Fisher's exact tests for categorical variables. *P*<0.05 was set as the threshold of statistical significance and marked in bold values. Abbreviations: BMI, body mass index. ^*^ Fisher's exact tests

**Supplementary Table 3** Mean EPDS scores with 95% confidence intervals by pregnancy stage and *hukou* status

| Characteristic | Pregnancy Stage | Mean Score | 95% CI |
| --- | --- | --- | --- |
| All | The first trimester | 3.54 | (3.36, 3.71) |
|  | The second trimester | 3.20 | (3.02, 3.37) |
|  | The third trimester | 3.65 | (3.47, 3.82) |
|  | Postpartum | 3.35 | (3.20, 3.50) |
| Non-local | The first trimester | 5.00 | (4.52, 5.49) |
| (*Hukou*) | The second trimester | 5.32 | (4.82, 5.82) |
|  | The third trimester | 5.39 | (4.97, 5.82) |
|  | Postpartum | 4.59 | (4.17, 5.02) |
| Local | The first trimester | 3.19 | (3.01, 3.38) |
| (*Hukou*) | The second trimester | 2.70 | (2.53, 2.88) |
|  | The third trimester | 3.24 | (3.06, 3.42) |
|  | Postpartum | 3.06 | (2.91, 3.21) |

Note: CI: confidence interval.

**Supplementary Table 4** Hosmer-Lemeshow Goodness-of-Fit Test results for logistic regression models across different gestational and postpartum stages

| Gestational and Postpartum Stages | Hosmer-Lemeshow χ² | df | *P* |
| --- | --- | --- | --- |
| The first trimester | 4.98 | 8 | 0.760 |
| The second trimester | 7.58 | 8 | 0.476 |
| The third trimester | 6.18 | 8 | 0.627 |
| Postpartum | 12.24 | 8 | 0.141 |

Note: A non-significant p-value (*P*>0.05) indicates adequate model fit, suggesting no significant discrepancy between observed and predicted outcomes. Degrees of freedom (df = 8) reflect the number of comparable groups formed after accounting for empty or tied-probability strata in the decile-based grouping.

**Supplementary Table 5** Results of multicollinearity analysis (variance Inflation Factor, VIF)

| Variable | VIF |
| --- | --- |
| Age | 1.235 |
| Pre-pregnancy BMI | 1.029 |
| Education | 1.317 |
| Only child | 1.083 |
| *hukou* | 1.232 |
| Marital status | 1.009 |
| Employment status | 1.054 |
| Household monthly income | 1.082 |
| Gravidity | 1.335 |
| HIV, syphilis, hepatitis B, and other infectious diseases | 1.013 |
| Planned pregnancy | 1.037 |

Note: Severe multicollinearity is defined as VIF > 10, and potential multicollinearity is defined as VIF > 5. VIF values for all independent variables are < 2, confirming the absence of multicollinearity.

**Supplementary Table 6** Multivariable logistic regression of depressive symptoms (EPDS≥9) related to *hukou* status and sociodemographic factors across pregnancy stages

| Characteristic | The first trimester | | | The second trimester | | | The third trimester | | | Postpartum | | |
| --- | --- | --- | --- | --- | --- | --- | --- | --- | --- | --- | --- | --- |
|  | aOR | 95% CI | *P* | aOR | 95% CI | *P* | aOR | 95% CI | *P* | aOR | 95% CI | *P* |
| *Hukou* status |  |  |  |  |  |  |  |  |  |  |  |  |
| Non-local | 1 |  |  | 1 |  |  | 1 |  |  | 1 |  |  |
| Local | 0.46 | (0.33, 0.63) | **<0.001** | 0.28 | (0.20, 0.38) | **<0.001** | 0.46 | (0.33, 0.65) | **<0.001** | 0.38 | (0.26, 0.55) | **<0.001** |
| Age group |  |  | 0.248 |  |  | 0.688 |  |  | 0.575 |  |  | **0.038** |
| <25 | 1 |  |  | 1 |  |  | 1 |  |  | 1 |  |  |
| 25-30 | 0.74 | (0.48, 1.14) | 0.172 | 0.77 | (0.49, 1.23) | 0.272 | 0.87 | (0.55, 1.39) | 0.566 | 0.75 | (0.45, 1.25) | 0.270 |
| 30-35 | 0.64 | (0.40, 1.01) | 0.055 | 0.82 | (0.50, 1.34) | 0.427 | 0.82 | (0.50, 1.33) | 0.418 | 0.73 | (0.42, 1.24) | 0.243 |
| ≥35 | 0.62 | (0.35, 1.09) | 0.094 | 0.72 | (0.39, 1.34) | 0.304 | 1.09 | (0.61, 1.95) | 0.768 | 0.31 | (0.14, 0.69) | **0.004** |
| Pre-pregnancy BMI group |  |  | 0.193 |  |  | 0.651 |  |  | 0.676 |  |  | 0.870 |
| <18.5kg/m2 | 1 |  |  | 1 |  |  | 1 |  |  | 1 |  |  |
| 18.5-24kg/m2 | 1.22 | (0.81, 1.84) | 0.354 | 1.03 | (0.67, 1.58) | 0.894 | 0.85 | (0.58, 1.26) | 0.421 | 1.03 | (0.62, 1.70) | 0.914 |
| ≥24kg/m2 | 1.51 | (0.95, 2.42) | 0.083 | 0.86 | (0.52, 1.44) | 0.573 | 0.82 | (0.52, 1.31) | 0.413 | 1.14 | (0.64, 2.03) | 0.663 |
| Education |  |  |  |  |  |  |  |  |  |  |  |  |
| High school or below | 1 |  |  | 1 |  |  | 1 |  |  | 1 |  |  |
| College or above | 0.74 | (0.53, 1.01) | 0.060 | 0.76 | (0.54, 1.09) | 0.134 | 0.95 | (0.68, 1.33) | 0.756 | 0.54 | (0.36, 0.81) | **0.003** |
| Only child |  |  |  |  |  |  |  |  |  |  |  |  |
| No | 1 |  |  | 1 |  |  | 1 |  |  | 1 |  |  |
| Yes | 0.48 | (0.35, 0.66) | **<0.001** | 0.75 | (0.54, 1.05) | 0.094 | 0.69 | (0.50, 0.94) | **0.018** | 0.76 | (0.52, 1.12) | 0.170 |
| Marital status |  |  |  |  |  |  |  |  |  |  |  |  |
| Divorced/unmarried | 1 |  |  | 1 |  |  | 1 |  |  | 1 |  |  |
| Married | 0.64 | (0.24, 1.69) | 0.370 | 0.87 | (0.28, 2.71) | 0.804 | 0.56 | (0.22, 1.47) | 0.241 | 1.36 | (0.30, 6.17) | 0.690 |
| Employment status |  |  | 0.908 |  |  | 0.666 |  |  | 0.273 |  |  | 0.658 |
| Unemployed | 1 |  |  | 1 |  |  | 1 |  |  | 1 |  |  |
| Freelance | 1.02 | (0.65, 1.60) | 0.935 | 0.80 | (0.49, 1.30) | 0.368 | 0.72 | (0.46, 1.13) | 0.157 | 0.78 | (0.44, 1.36) | 0.378 |
| Full-time | 1.08 | (0.74, 1.55) | 0.700 | 0.90 | (0.61, 1.32) | 0.585 | 0.77 | (0.53, 1.10) | 0.143 | 0.85 | (0.55, 1.32) | 0.471 |
| Household monthly income |  |  | 0.809 |  |  | 0.398 |  |  | 0.603 |  |  | 0.230 |
| <10000RMB | 1 |  |  | 1 |  |  | 1 |  |  | 1 |  |  |
| 10000-20000RMB | 1.10 | (0.81, 1.48) | 0.546 | 1.04 | (0.75, 1.44) | 0.827 | 0.91 | (0.67, 1.24) | 0.553 | 1.39 | (0.95, 2.03) | 0.094 |
| ≥20000RMB | 1.10 | (0.74, 1.65) | 0.629 | 1.32 | (0.87, 2.01) | 0.198 | 1.10 | (0.74, 1.64) | 0.640 | 1.13 | (0.67, 1.91) | 0.649 |
| Gravidity |  |  | 0.249 |  |  | 0.988 |  |  | 0.231 |  |  | 0.094 |
| 1 | 1 |  |  | 1 |  |  | 1 |  |  | 1 |  |  |
| 2-4 | 1.26 | (0.93, 1.72) | 0.142 | 0.99 | (0.71, 1.38) | 0.961 | 1.20 | (0.87, 1.65) | 0.263 | 0.85 | (0.58, 1.26) | 0.414 |
| ≥5 | 1.53 | (0.83, 2.85) | 0.177 | 1.04 | (0.52, 2.08) | 0.908 | 1.69 | (0.91, 3.12) | 0.097 | 1.72 | (0.83, 3.58) | 0.147 |
| HIV, syphilis, hepatitis B, and other infectious diseases |  |  |  |  |  |  |  |  |  |  |  |  |
| No | 1 |  |  | 1 |  |  | 1 |  |  | 1 |  |  |
| Yes | 2.31 | (1.31, 4.07) | **0.004** | 3.01 | (1.69, 5.35) | **<0.001** | 1.92 | (1.07, 3.43) | **0.029** | 3.52 | (1.85, 6.69) | **<0.001** |
| Planned pregnancy |  |  |  |  |  |  |  |  |  |  |  |  |
| No | 1 |  |  | 1 |  |  | 1 |  |  | 1 |  |  |
| Yes | 0.75 | (0.56, 0.99) | **0.041** | 1.02 | (0.74, 1.39) | 0.922 | 0.98 | (0.73, 1.32) | 0.882 | 0.68 | (0.48, 0.96) | **0.030** |

Note: EPDS = Edinburgh Postnatal Depression Scale; aOR = adjusted odds ratio; CI = confidence interval. All models adjusted for age, education, pre-pregnancy BMI, only child, *hukou* status, marital status, employment, household monthly income, gravidity, HIV, syphilis, hepatitis B, and other infectious diseases, and planned pregnancy. *P* < 0.05 was set as the threshold of statistical significance and marked in bold values.

**Supplementary Table 7** Longitudinal GEE analyses of three *hukou*-based SES interactions on perinatal depressive symptoms (N = 7,300 observations)

| Characteristic | β | SE | Wald χ² | *P* | aOR | 95%CI |
| --- | --- | --- | --- | --- | --- | --- |
| *hukou*×education | −0.031 | 0.254 | 0.01 | 0.903 | 0.97 | (0.59, 1.60) |
| *hukou*×employment status | 0.063 | 0.146 | 0.19 | 0.663 | 1.07 | (0.80, 1.43) |
| *hukou*×household monthly income | −0.195 | 0.172 | 1.29 | 0.256 | 0.82 | (0.59, 1.15) |

Note: SE = Standard Error; aOR = adjusted odds ratio; 95%CI = 95% Confidence Interval; GEE = Generalized Estimating Equations. Three separate GEE models (exchangeable correlation) were fitted to test (i) *hukou* × education, (ii) *hukou* × household monthly income, and (iii) *hukou* × employment interactions. All models included *hukou* status, time and *hukou* ×time interactions as covariates and were adjusted for age, education, pre-pregnancy BMI, only-child status, marital status, employment, household monthly income, gravidity, HIV, syphilis, hepatitis B, other infectious diseases, and planned pregnancy.

Within-subject correlation estimates: α (SE) = 0.296 (0.047), 0.296 (0.047), and 0.294 (0.043) for models (i), (ii), and (iii), respectively.

Time variable assignment: 1 = first trimester, 2 = second trimester, 3 = third trimester, 4 = postpartum period.

**Supplementary Table 8** Incidence of depressive symptoms across perinatal stages under different EPDS cutoff values

|  | The first trimester | The second trimester | The third trimester | Postpartum |
| --- | --- | --- | --- | --- |
| EPDS≥9 | 15.62% | 12.99% | 14.08% | 9.15% |
| EPDS≥10 | 11.0% | 8.55% | 9.75% | 5.75% |
| EPDS≥13 | 3.18% | 2.30% | 2.63% | 1.37% |

Note:  EPDS = Edinburgh Postnatal Depression Scale.

**Supplementary Table 9**　Longitudinal GEE analyses of the association between *hukou* status and perinatal depression under different EPDS cutoff values (N = 7,300 observations)

| EPDS cutoff values |  |  | β | SE | Wald χ² | *P* | aOR | 95%CI |
| --- | --- | --- | --- | --- | --- | --- | --- | --- |
| EPDS≥10 | Model1 | *hukou* | −1.135 | 0.195 | 33.96 | **<0.001** | 0.32 | (0.24, 0.44) |
|  |  | time | -0.144 | 0.045 | 10.08 | **0.002** | 0.87 | (0.80, 0.94) |
|  |  | *hukou*×time | -0.072 | 0.063 | 1.32 | 0.251 | 0.93 | (0.83, 1.05) |
|  | Model2 | *hukou* | -0.832 | 0.212 | 15.36 | **<0.001** | 0.44 | (0.30, 0.63) |
|  |  | time | -0.148 | 0.047 | 10.12 | **0.002** | 0.86 | (0.79, 0.94) |
|  |  | *hukou*×time | -0.071 | 0.064 | 1.22 | 0.269 | 0.93 | (0.82, 1.06) |
| EPDS≥13 | Model1 | *hukou* | -1.422 | 0.345 | 16.99 | **<0.001** | 0.24 | (0.12, 0.48) |
|  |  | time | -0.185 | 0.083 | 4.93 | **0.026** | 0.83 | (0.71, 0.98) |
|  |  | *hukou*×time | -0.087 | 0.123 | 0.51 | 0.475 | 0.92 | (0.72, 1.17) |
|  | Model2 | h*ukou* | -1.185 | 0.383 | 9.59 | **0.002** | 0.31 | (0.14, 0.65) |
|  |  | time | -0.188 | 0.084 | 4.94 | **0.026** | 0.83 | (0.70, 0.98) |
|  |  | *hukou*×time | -0.086 | 0.123 | 0.48 | 0.487 | 0.92 | (0.72, 1.17) |

Note: EPDS = Edinburgh Postnatal Depression Scale; SE = Standard Error; aOR = adjusted odds ratio; 95%CI = 95% Confidence Interval; GEE = Generalized Estimating Equations. Model 1 included *hukou* status, time and *hukou*×time as the variable. Model 2 adjusted for age, education, pre-pregnancy BMI, only child, marital status, employment, household monthly income, gravidity, HIV, syphilis, hepatitis B, and other infectious diseases, and planned pregnancy.

For EPDS ≥ 10: Model 1 intercept (α) = 0.260 (SE = 0.057); Model 2 intercept (α) = 0.232 (SE = 0.061).​For EPDS ≥ 13: Model 1 intercept (α) = 0.186 (SE = 0.193); Model 2 intercept (α) = 0.145 (SE = 0.197).

Time variable assignment: 1 = first trimester, 2 = second trimester, 3 = third trimester, 4 = postpartum period.

*P*<0.05 was set as the threshold of statistical significance and marked in bold values.

**Supplementary Table 10** Logistic regression analysis of the association between *hukou* status and perinatal depression under different EPDS cutoff values

|  | The first trimester | | The second trimester | | The third trimester | | Postpartum | |
| --- | --- | --- | --- | --- | --- | --- | --- | --- |
|  | aOR | 95% CI | aOR | 95% CI | aOR | 95% CI | aOR | 95% CI |
| EPDS ≥ 10 |  |  |  |  |  |  |  |  |
| Model1 |  |  |  |  |  |  |  |  |
| Non-local | 1 |  | 1 |  | 1 |  | 1 |  |
| Local | 0.37 | (0.27, 0.51) ^***^ | 0.16 | (0.11, 0.22) ^***^ | 0.38 | (0.27, 0.53) ^***^ | 0.21 | (0.14, 0.31) ^***^ |
| Model2 |  |  |  |  |  |  |  |  |
| Non-local | 1 |  | 1 |  | 1 |  | 1 |  |
| Local | 0.55 | (0.38, 0.79) ^**^ | 0.21 | (0.14, 0.31) ^***^ | 0.48 | (0.33, 0.71) ^*^ | 0.29 | (0.18, 0.46) ^***^ |
| EPDS ≥ 13 |  |  |  |  |  |  |  |  |
| Model1 |  |  |  |  |  |  |  |  |
| Non-local | 1 |  | 1 |  | 1 |  | 1 |  |
| Local | 0.27 | (0.16, 0.46) ^***^ | 0.10 | (0.05, 0.19) ^***^ | 0.31 | (0.17, 0.56) ^***^ | 0.13 | (0.06, 0.29) ^***^ |
| Model2 |  |  |  |  |  |  |  |  |
| Non-local | 1 |  |  |  |  |  |  |  |
| Local | 0.39 | (0.22, 0.72) ^**^ | 0.12 | (0.06, 0.25) ^***^ | 0.31 | (0.17, 0.58) ^***^ | 0.23 | (0.10, 0.54) ^***^ |

Note: EPDS = Edinburgh Postnatal Depression Scale; aOR = adjusted odds ratio; CI = confidence interval. EPDS ≥ 10: use standard logistic regression; EPDS ≥ 13: use Firth logistic regression. Model 1 included *hukou* status as the sole variable. Model 2 adjusted for age, education, pre-pregnancy BMI, only child, marital status, employment, household monthly income, gravidity, HIV, syphilis, hepatitis B, and other infectious diseases, and planned pregnancy.

Significance levels: ^*^*P*<0.05; ^**^*P*<0.01; ^***^*P*<0.001.


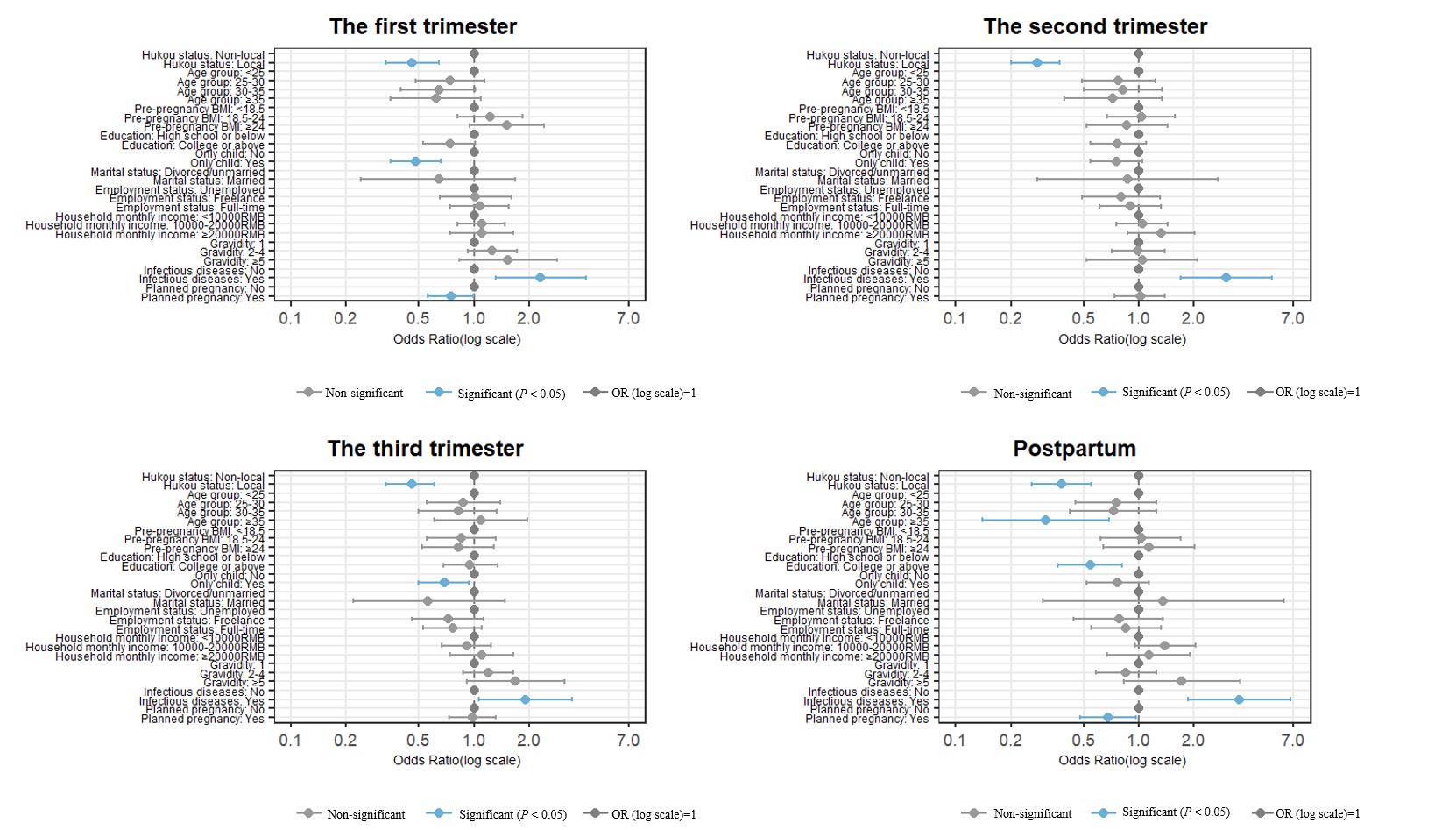


**Supplementary Fig. 1** Forest plot analysis of *hukou* status and covariates associated with depression symptoms across pregnancy stages (multivariate logistic regression)
